# A Multidisciplinary Approach to Obesity Hypoventilation Syndrome: From Diagnosis to Long-Term Management—A Narrative Review

**DOI:** 10.3390/diagnostics15172120

**Published:** 2025-08-22

**Authors:** Mara Andreea Vultur, Bianca Liana Grigorescu, Dragoș Huțanu, Edith Simona Ianoși, Corina Eugenia Budin, Gabriela Jimborean

**Affiliations:** 1 Pulmonology Department, George Emil Palade University of Medicine, Pharmacy, Science, and Technology of Târgu Mureș, 540139 Târgu Mureș, Romania; mara.vultur@umfst.ro (M.A.V.); edith.ianosi@umfst.ro (E.S.I.); gabriela.jimborean@umfst.ro (G.J.); 2 Doctoral School of Medicine and Pharmacy, George Emil Palade University of Medicine, Pharmacy, Science, and Technology of Târgu Mureș, 540139 Târgu Mureș, Romania; 3 Pulmonology Clinic, Mureș County Clinical Hospital, 540011 Târgu Mureș, Romania; 4 Anesthesiology and Intensive Care Department, George Emil Palade University of Medicine, Pharmacy, Science, and Technology of Târgu Mureș, 540139 Târgu Mureș, Romania; 5 Pathophysiology Department, George Emil Palade University of Medicine, Pharmacy, Science, and Technology of Târgu Mureș, 540139 Târgu Mureș, Romania; corina.budin@umfst.ro

**Keywords:** obesity-hypoventilation syndrome, critical care, sleep apnea, positive-airway pressure, diagnostic scores

## Abstract

Obesity Hypoventilation Syndrome (OHS), also known as Pickwickian syndrome, is a complex disorder characterized by obesity (BMI > 30 kg/m^2^), daytime hypercapnia (PaCO_2_ ≥ 45 mmHg), and sleep-disordered breathing, primarily affecting individuals with severe obesity. Its diagnosis requires the exclusion of other causes of alveolar hypoventilation and involves comprehensive assessments, including clinical history, physical examination, pulmonary function tests, arterial blood gases, and sleep studies. The pathophysiology of OHS involves mechanical constraints from excessive adipose tissue, diminished central respiratory drive often linked to leptin resistance, mitochondrial dysfunction, and oxidative stress, all contributing to impaired ventilation and systemic inflammation. The condition often coexists with obstructive sleep apnea (OSA), exacerbating nocturnal hypoxia and hypercapnia, which can lead to severe cardiopulmonary complications such as pulmonary hypertension and right-sided heart failure. Epidemiologically, the rising global prevalence of obesity correlates with an increased incidence of OHS, yet underdiagnosis remains a significant challenge, often resulting in critical presentations like acute hypercapnic respiratory failure. Management primarily centers on non-invasive ventilation modalities like CPAP and BiPAP, with an emphasis on individualized treatment plans, continuous monitoring, and addressing comorbidities such as hypertension and diabetes. Pharmacological interventions are still evolving, focusing on supportive care and metabolic regulation. Long-term adherence, psychological factors, and complications like ventilator failure or device intolerance highlight the need for ongoing multidisciplinary management. Overall, advancing our understanding of OHS’s multifactorial mechanisms and optimizing tailored therapeutic strategies are crucial for improving patient outcomes and reducing mortality associated with this increasingly prevalent syndrome.

## 1. Introduction

Obesity-hypoventilation syndrome (OHS), also referred to as Pickwickian syndrome, is a disorder characterized by the presence of obesity (body mass index (BMI) > 30 kg/m^2^), daytime hypercapnia (arterial carbon dioxide (PaCO_2_) ≥ 45 mmHg) during wakefulness, and sleep disordered breathing (SDB). The diagnosis is established after ruling out other causes of alveolar hypoventilation, such as severe obstructive or restrictive pulmonary diseases, medication, drugs and alcohol abuse, neuromuscular and skeletal disorders, i.e., low skeletal muscle strength occurring in geriatric syndromes like sarcopenia, or other central hypoventilation syndromes related to central nervous system infections, brain tumors, trauma or sequelae of neurological procedures [1,2]. Also, differentiating OHS from sleep hypoventilation is crucial in clinical practice due to their overlapping features, despite differing underlying pathophysiology and clinical presentations. Sleep hypoventilation generally denotes episodes of reduced ventilation occurring solely during sleep, without a necessary association with obesity or persistent hypercapnia during the day. Accurately distinguishing between these conditions is essential for appropriate diagnosis and targeted management [3]. Several studies have also suggested including a calculated arterial standard or venous serum bicarbonate (HCO^3−^) into the definition of OHS, which, in the absence of another influence on metabolic acid-base status, would represent a 24-h guide to a patient’s ventilation. A single one-off measurement of PaCO_2_ is thought to be influenced by factors like the ability of some obese patients who hypoventilate overnight to return their PaCO_2_ within normal values a short time following awakening. Also, studies have reported that increased serum bicarbonate may also suggest multimorbidity and polypharmacy, which have to be taken into consideration in the process of OHS management [1,3]. Unrecognized and untreated OHS represents a powerful risk factor for the adverse outcomes of the critically ill patient, with increased morbidity and mortality rates [4].

## 2. Epidemiological Insights

The worldwide incidence of obesity is rising rapidly, impacting an estimated 2.8 million people each year according to recent data [5]. In 2020, more than 650 million adults globally were classified as obese (BMI ≥ 30 kg/m^2^), highlighting a major public health challenge [6]. Additionally, concerns are growing about the increasing rates of childhood obesity, with predictions suggesting that in 2020, around 158 million children and adolescents aged 5 to 19 are affected, a number projected to reach 254 million by 2030 [7].

The prevalence of OHS is a growing concern, with estimates suggesting that around 0.4% of adults are affected. The condition appears more frequently among individuals with severe obesity, highlighting the significant health risks associated with extreme weight [8]. Studies reveal that around 90% of individuals with OHS also have obstructive sleep apnea (OSA), highlighting a significant overlap between these two disorders [9]. The growing rates of obesity within the broader population are closely linked to the upward trend in OHS cases observed in critical care settings [10]. Additionally, the connection between obesity and hypoventilation syndromes significantly elevates the risk of developing complications, including pulmonary hypertension and right-sided heart failure. Nearly half of the patients with OHS may need mechanical ventilation, highlighting the serious and potentially life-threatening nature of this condition [11].

Patients with OHS are often underdiagnosed in the outpatient setting, which can lead to delayed recognition and management of the condition. Consequently, this often leads to their initial hospital presentation with severe respiratory complications. Notably, acute hypercapnic respiratory failure (AHRF) is the leading cause of admission to the ICU for approximately 40% of OHS patients, followed by hypoxemic respiratory failure in 63% of cases [12,13]. Additional causes for admission include sepsis cases (41%), cardiac heart failure in 39% of cases, depressant medications (5%), trauma and surgery (each in 3% of cases), but only 10% of patients had a certain diagnosis of OHS at the moment of discharge [9,14].

## 3. Pathophysiological Patterns

At the core of the pathophysiology of OHS is the accumulation of excess adipose tissue around the thoracic and abdominal regions, which can significantly compromise respiratory mechanics [15]. This condition results in reduced lung volumes and heightened airway resistance, primarily due to external compression of the airways. The increased thoracic circumference significantly limits lung compliance and restricts thoracic expansion necessary for optimal ventilation. Consequently, higher intrathoracic pressure during inhalation further exacerbates airway resistance, impairing the efficiency of air exchange [16]. The presence of adipose tissue can induce a chronic, low-grade inflammatory state contributing to edema in the airways, further complicating respiratory mechanics and making it increasingly difficult for patients to maintain adequate ventilation [17].

Additionally, individuals with OHS typically experience a diminished central respiratory drive, making them more susceptible to respiratory failure during sleep or periods of sedation [18]. This diminished sensitivity is hypothesized to stem from leptin resistance, a condition wherein the normal signaling pathway of leptin—a hormone intimately tied to energy homeostasis—becomes disrupted, compromising the stimulation of the respiratory centers [19,20]. Furthermore, a key factor in the complex nature of OHS is leptin’s impact on the hypothalamic regulation of respiratory distress. This hormonal imbalance interacts with autonomic control systems and respiratory reflexes, highlighting how leptin resistance can exacerbate the clinical manifestations of OHS by sustaining a cycle of hypoventilation and respiratory instability [4]. To optimize treatment outcomes for patients with OHS and enhance their respiratory function, it is crucial to address both the correction of this maladaptive hormonal response and the underlying mechanical factors.

Mitochondria play a crucial role in regulating cellular energy processes, and their impaired function is commonly linked to obesity and associated metabolic conditions. Research has shown that disruptions in mitochondrial activity can result in reduced adiponectin production and heightened insulin resistance, both of which are key contributors to the development of obesity-related health issues [21], ultimately mediating critical disease processes, including sepsis, acute lung injury, acute renal failure, and ICU-skeletal muscle dysfunction [22,23]. Within the framework of OHS, mitochondrial dysfunction potentially plays a pivotal role in the disease process by impacting cellular energy regulation, respiratory regulation, and systemic inflammatory responses. Mitochondria’s role in controlling reactive oxygen species (ROS) production is crucial. In obesity, heightened fatty acid oxidation elevates ROS levels, causing oxidative stress that can damage cells and impair brain regions governing respiration, potentially worsening hypoventilation [24]. The link between ROS and respiratory regulation is significant, as oxidative stress can impair chemoreceptor function in the brain, reducing sensitivity to hypercapnia and hypoxia [25]. Mitochondrial uncoupling proteins (UCPs) play a key role in energy regulation and thermogenesis. Their dysregulation may connect obesity with respiratory issues, reducing energy metabolism and promoting fat gain, which worsens the hypercapnia and hypoxia seen in OHS [26].

Another complicating factor is the common presence of obstructive sleep apnea (OSA) in individuals with OHS, which can worsen respiratory dysfunctions during the night. This connection results in periodic airway blockages, leading to intermittent hypoxia and increased levels of hypercapnia [27]. Nevertheless, in patients with obesity hypoventilation syndrome, these mechanisms are frequently impaired or diminished owing to the coexistence of obesity and metabolic disturbances. As a result, individuals are often unable to effectively respond to progressively elevated carbon dioxide levels or diminished oxygen saturation during sleep, which can lead to sustained hypoventilation and intensify the severity of daytime hypercapnia [28]. Furthermore, the interaction between obesity and obstructive sleep apnea in the context of OHS underscores the significant cardiovascular challenges these conditions present. Frequent hypoxic episodes stemming from both disorders induce oxidative stress and stimulate inflammatory pathways, which can increase the risk of developing hypertension and ultimately contribute to heart failure [20].

A deeper view of the onset mechanisms of obesity hypoventilation syndrome (OHS) reveals that endothelial dysfunction and chronic inflammation are fundamental components of obesity pathophysiology, playing a central role in the multiple decompensations that often lead to intensive care unit admissions [29]. The primary focus regarding the critically ill patient with obesity is directed towards advanced hemodynamic and respiratory support, renal dysfunction, and neurological dysregulation. Attention should also be placed upon maintaining endocrine stability, given the interplay that arises between inflammation, coagulopathy, and hormonal dysregulation in the critical setting [30].

## 4. Initial Assessment

The initial evaluation of patients suspected of obesity hypoventilation syndrome is vital for precise diagnosis and effective treatment planning. Recognizing this condition early depends on a deep understanding of the complex interplay between obesity, respiratory dysfunction, and clinical signs. The process usually starts with a comprehensive medical history, focusing on weight trends, symptom patterns, and coexisting health issues. Notably, a significant rise in BMI, especially when coupled with symptoms like excessive daytime sleepiness, fatigue, and cognitive challenges, often signals clinicians to consider OHS as a probable diagnosis [16,27]. The STOP-BANG questionnaire is a validated screening tool tailored to identify individuals at high risk for obstructive sleep apnea, a condition commonly co-occurring with OHS. Since OSA can intensify hypoventilation and complicate clinical management, effective screening through this instrument is essential for early detection and comprehensive treatment planning, ultimately improving patient outcomes [31].

Beyond clinical history, a thorough physical exam is essential in the initial assessment of suspected OHS. Obesity is often visibly prominent, accompanied by signs of respiratory difficulty or reduced breath sounds. Features such as a thick neck circumference can hint at obstructive patterns during sleep, raising suspicion for obstructive sleep apnea (OSA). Additionally, vital signs—especially oxygen saturation and respiratory rate—must be carefully evaluated, as hypoxemia signals substantial respiratory impairment that warrants prompt intervention [32].

Pulmonary function tests (PFTs) and arterial blood gas (ABG) analyses form the cornerstone of initial OHS assessment. PFTs provide vital insights into airflow dynamics and lung volumes, while ABG measurements reveal the extent of systemic gas exchange impairment. Notably, a PaCO_2_ exceeding 45 mmHg is a key indicator of hypoventilation. These tests often display a restrictive lung profile or hypoventilation signs, helping clinicians distinguish OHS from other conditions like chronic obstructive pulmonary disease (COPD) and confirming the diagnosis with precision [2].

In addition, comprehensive sleep studies such as polysomnography are essential for diagnosing OHS in patients suspected of having sleep-related breathing disorders. These overnight assessments meticulously monitor breathing patterns, pinpointing apnea-hypopnea events, measuring maximum desaturation episodes, and revealing hypoventilation patterns that occur during sleep. Such detailed insights are crucial in confirming the presence of OHS and guiding targeted interventions [16,25]. Understanding sleep patterns sheds light on the link between hypoventilation and nighttime desaturation, helping to gauge disease severity and its impact on overall health.

A collaborative effort among pulmonologists, endocrinologists, and metabolic experts enriches diagnosis and addresses key complications. Early specialist involvement is especially vital for patients with overlapping features of OHS and ROHHAD, ensuring more effective management [33,34].

The primary objective for patients with OHS who are admitted to the ICU is the prevention of respiratory complications and multiple organ failure. Different evaluation and predicting scores can offer a dynamic view of the treatment approach and outcomes, but there is still no validated score for predicting the severity and mortality risk of obese patients. Studies have shown a J-shaped relationship between BMI and mortality among hospitalized and ICU patients, as well as those with chronic illnesses, where being overweight or having moderate obesity appears to offer some protection compared to having a normal BMI or more severe obesity—a phenomenon often referred to as the “obesity paradox,” which remains a topic of ongoing debate and incomplete understanding [35,36]. Current severity scoring systems might inaccurately assess patients by failing to adequately consider the physiological variations associated with obesity, which could result in an underestimation of the actual risk [37]. The final score may be influenced by the abnormal arterial blood gas results, such as those seen in minor pneumonia with low oxygen delivery (DO_2_) and decreased alveolar arterial (A-a) gradient, which indicates impaired oxygen exchange in the lungs and may contribute to an artificially higher APACHE II score. In the same manner, low urine output in some obese patients will increase the final score due to overestimation of renal dysfunction [38].

ICU scoring systems traditionally employed to evaluate the severity and prognosis of critically ill patients often fail to address the needs of obese individuals specifically. Therefore, it is essential to consider detailed mortality prognostic scores for this group. Obese patients face higher risks for complications like hypoventilation and obstructive sleep apnea, which complicate their management in the ICU. Studies show that obesity is linked to extended durations of mechanical ventilation and increased challenges in weaning, attributed to factors such as elevated airway resistance and diaphragm displacement [39]. OHS further elevates the risk of respiratory failure and poses challenges in managing ventilation within the ICU. Studies indicate that a substantial proportion of surgical patients, including individuals undergoing bariatric surgeries, present with high STOP-BANG scores—corresponding strongly with the presence of OSA [40]. Thus, serving different purposes, they may be valuable in the screening process of critically ill OHS patients admitted to the ICU. Intertwined features of both APACHE II and STOP-BANG scores are the age and blood pressure (BP), the increasing age—above 50 years old—having a negative impact on the final outcomes. The mean BP evaluated by the APACHE II score is better at stratifying the severity and risks than the STOP-BANG score, which only considers hypertension a risk factor in developing obstructive sleep apnea. The BMI evaluation of the STOP-BANG score can be the missing puzzle piece of ICU predicting scores, changing the approach to management for a BMI > 35 kg/m^2^. The analysis of the STOP-BANG questionnaire and the ICU predicting scores unveils a noteworthy connection between an increased risk of obstructive sleep apnea (STOP-BANG score ≥ 3) and heightened probabilities of experiencing acute respiratory failure, electrolyte imbalance, multiple organ failure and death [31,41,42], emphasizing the need for extensive multicentered studies to develop a key score in the management of those patients.

## 5. Respiratory Management

Major components taken into consideration regarding the therapeutic interventions for OHS patients include: the use of positive airway pressure therapy (PAP), oxygen supplementation, pharmacological treatment, assessment of comorbidities, and weight reduction treatment. While the morbidity and mortality rates among critically ill patients diagnosed with OHS are substantial, it is imperative that the management of OHS addresses both the immediate concerns, such as acute respiratory failure, as well as the long-term considerations encompassing obesity, chronic respiratory failure, and associated comorbidities [43,44,45,46,47].

After establishing both OHS and co-existing OSA diagnoses, addressing respiratory insufficiency predominantly involves non-invasive ventilation techniques, with continuous positive airway pressure (CPAP) and bi-level positive airway pressure (BiPAP) serving as the main therapeutic options [48]. Continuous positive airway pressure (CPAP) demonstrates efficacy in stabilizing patients with OHS and obstructive sleep apnea by addressing various physiological mechanisms. It eliminates upper airway obstruction, enhances alveolar recruitment, and augments functional residual capacity (FRC) [12,49,50]. Additionally, it counteracts intrinsic positive end-expiratory pressure (PEEP), relieves strain on respiratory muscles, diminishes the work of breathing (WOB), and enhances ventilation/perfusion matching [51]. However, the use of CPAP is discouraged in acute scenarios, as it is unlikely to alleviate alveolar hypoventilation issues in acutely ill patients with obesity hypoventilation syndrome who present with acute hypercapnic respiratory failure [52].

Non-invasive positive pressure ventilation (NPPV) serves as a vital treatment approach for individuals suffering from OHS, especially during acute respiratory failure episodes. This intervention plays a key role in stabilizing patients by reducing upper airway obstruction, increasing alveolar recruitment, and boosting FRC. As noted by Bry et al., the initial use of NPPV can significantly enhance gas exchange. Patients with a higher BMI often require elevated levels of inspiratory positive airway pressure (IPAP) due to the heightened pleural pressure experienced by those who are obese [53,54]. The NPPV initiation process in obese patients with AHRF should primarily optimize body position to improve respiratory compliance and gas exchange [52]. Some strategies may include sitting position, reverse Trendelenburg, and “beach chair position”, being used even during laparoscopic surgeries in obese patients [55]. Prone position may also help alleviate the end-expiratory lung volume (EELV) and gas exchange in mechanically ventilated patients, compared to the supine position [56,57]. The variation in breathing patterns observed with different ventilators specifically addresses the challenges posed by excess body fat, which impacts airway dynamics and gas exchange. In critical care settings, adopting tailored respiratory approaches that incorporate ideal PEEP levels, recruitment maneuvers, and appropriate ventilatory modes offers improved results in the management of patients with Obesity Hypoventilation Syndrome [58,59]. Research indicates that around 84% of individuals with AHRF achieve favorable results with NPPV. However, the remaining 16% may encounter complications that necessitate intubation or, in some cases, result in mortality. Factors influencing these outcomes often include the initial severity of the condition and how well the patient responds to the employed treatment strategies [53,59]. Delayed failure of non-invasive ventilation (NIV), characterized by the development of acute hypercapnic respiratory failure with respiratory acidosis occurring beyond 48 h after initial stabilization, poses considerable clinical difficulties. This late-onset complication is commonly seen in patients with acidotic OHS, especially among those who have been administered diuretics or medications prior to hospital admission that may contribute to respiratory depression [60].

Extensive research underscores the importance of meticulous respiratory mechanics monitoring in patients with OHS to optimize clinical outcomes. Leptin influences pulmonary surfactant production, critical for alveolar stability. In OHS, elevated leptin and inflammation will increase oxidative stress, damaging epithelial tissues and impairing surfactant synthesis [61]. This disruption heightens vulnerability to lung injuries such as ventilator-induced lung injury (VILI) and patient self-inflicted lung injury (PSILI), as inflammation and oxidative damage weaken alveolar integrity and exacerbate lung injury risks. Tracking esophageal and transpulmonary pressures is vital for tailoring protective ventilatory strategies, such as PAP therapy, which can substantially decrease the risk of pulmonary surfactant damage and VILI [62]. Neglecting these measurements may result in suboptimal ventilation adjustments, exacerbating respiratory impairments [63]. Managing sleep apnea effectively in obese OHS patients necessitates careful calibration of ventilatory settings to prevent worsening gas exchange and reduce apnea episodes [64]. Integrating pressure metrics with arterial blood gas analyses provides real-time data, guiding precise ventilation modifications that promote adequate oxygenation while safeguarding against additional lung injury [65].

Invasive mechanical ventilation treatment in obesity hypoventilation syndrome patients has to be taken into consideration in cases of NPPV failure. Features like persistent pH levels < 7.25, low oxygen levels, hypotension, bradycardia, persistent dyspnea and tachypnea, high accessory muscle use, mental status decline, and psychomotor agitation may suggest the need for intubation [66,67]. Morbidly obese patients with acute hypercapnic respiratory failure pose challenges for the intubation process in over 50% of cases, even with experienced critical care specialists, with at least three attempts before successful intubation [68].

Intubation maneuvers should always be considered challenging in obese patients, taking into consideration older age, higher BMI, and higher Mallampati score. Research conducted in both operating room and ICU settings indicates that a modified Mallampati class III or IV, commonly linked to obesity, serves as a more significant independent predictor of difficult intubation than BMI itself [69,70]. Patient preparation is the key to a successful intubation, with prolonged time-to-desaturation, due to the rapid drop of FRC during sedation [71,72].

Research by Sakaguchi et al. suggests that combining high-flow nasal cannula (HFNC) therapy with optimal patient positioning offers notable advantages, proposing it as a viable alternative to traditional non-invasive ventilation, particularly for high-risk and obese individuals undergoing anesthesia [73]. Similarly, HFNC’s effectiveness in maintaining adequate oxygenation during perioperative care in patients with hypoxemic respiratory failure [74]. Badiger et al. also emphasized the benefits of HFNC in improving intubation conditions, while enhancing patient comfort and compliance compared to standard oxygen delivery methods, as well as decreasing the length of admission [75,76]. However, potential challenges such as barotrauma risk and limitations in certain clinical situations underscore the importance of close monitoring and tailored treatment approaches when implementing HFNC therapy [77,78].

## 6. Medication and Long-Term Management

The management of OHS is inherently multidisciplinary, requiring the collaboration of pulmonologists, sleep medicine experts, dietitians, cardiologists, psychiatrists, and primary and intensive care physicians. This collective approach is vital to tackling the complex, interconnected challenges of the syndrome. Achieving optimal outcomes hinges on a balanced integration of pharmacological treatments, lifestyle modifications, and behavioral interventions addressing psychological barriers, ensuring that each patient receives personalized, holistic care tailored to their unique needs [79,80,81].

The pharmacological approach to treating OHS is still in its formative stages, with ongoing developments. Currently, management predominantly emphasizes supportive therapies, lifestyle adjustments, and addressing associated health conditions, rather than employing drugs that are specifically tailored for OHS itself [2,32]. Instead, therapeutic efforts are directed toward controlling comorbid conditions like hypertension, diabetes, and dyslipidemia. Consequently, medications such as antihypertensives and antidiabetic agents are frequently utilized, with their primary benefit deriving from enhancing metabolic health, which in turn can positively influence respiratory function [1,82]. While bronchodilators have been employed in cases where bronchospasm exacerbates OHS, their use should be carefully guided by pulmonary function assessments and clinical evaluation to ensure appropriate and effective management [2]. The potential of behavioral pharmacological strategies, such as appetite suppressants or metabolic agents, as supportive tools to enhance adherence to weight loss efforts is emphasized by the latest research. These interventions should be tailored to each patient’s unique medical history and clinical needs to maximize safety and efficacy [1,83].

Standard medication dosing protocols are frequently derived from studies involving individuals with normal body weight, which raises concerns about their relevance for obese patients, as differences in drug clearance and volume of distribution (Vd) can be significant. Many weight-based dosing recommendations lack clarity on whether to use actual body weight, ideal weight (calculated from height and sex), or adjusted weight (a compromise between the two), and they often overlook other obesity-related factors vital for accurate dosing [84,85]. Additionally, obesity-related conditions such as diabetes can cause glomerular hyperfiltration in some patients, while others with chronic kidney disease (CKD) or acute kidney injury (AKI) may experience reduced glomerular filtration rates [84,85]. This wide variability complicates the process of determining appropriate doses for medications eliminated through the kidneys in obese individuals [85].

Updated guidelines on managing pain, agitation, sedation, delirium, immobility, and sleep disturbances in critically ill patients (PADIS) highlight the importance of creating evidence-based strategies that are adapted to diverse patient groups, including those with obesity [86]. When it comes to medication dosing in obese ICU patients, significant debate persists, as pharmacokinetic and pharmacodynamic alterations are common due to increased body weight and changes in drug clearance, impacting both safety and efficacy [87]. For instance, sedatives such as propofol and dexmedetomidine often depend on actual body weight or adjusted weight for dosing, whereas drugs like midazolam may require more nuanced titration—initially calculated based on weight but adjusted according to patient response to reach the target sedative effect [88]. Additionally, excessive or prolonged sedation has been linked to complications such as delayed awakening and extended ventilation times, emphasizing the importance of cautious and individualized dosing strategies in this vulnerable population [87].

Dexmedetomidine has emerged as a highly favored sedative choice for managing patients with OHS in the ICU setting, owing to its distinctive pharmacological properties that facilitate sedation without significantly suppressing respiratory function [89,90]. Research shows that as an α2-adrenergic receptor agonist, dexmedetomidine not only provides effective sedation but also improves patient comfort and compliance with non-invasive ventilation (NIV), which may lead to shorter ICU stays and better clinical outcomes [91,92]. In cases where airway management proves difficult, particularly when patients exhibit ongoing respiratory distress or agitation, dexmedetomidine can be used as an adjunct to traditional sedatives like propofol and midazolam, helping to lower overall sedative doses and increase the number of ventilator-free hours [91,93]. Its capacity to minimize the hemodynamic side effects often associated with other sedatives while preserving a stable respiratory drive underscores its utility in the care of critically ill obese patients [94]. In a study examining the pharmacokinetics and pharmacodynamics of dexmedetomidine in morbidly obese individuals versus non-obese patients undergoing laparoscopic procedures, Bo Xu and colleagues found that sedation was deeper and oxygen saturation levels were notably lower in the obese group [95]. Emphasized adverse effects of dexmedetomidine, such as bradycardia, hypotension, or hypertension, should be carefully monitored, especially in ICU patients with unstable hemodynamics or existing heart conditions [96].

Treating pain in individuals with OHS necessitates cautious opioid administration, given the heightened risk of opioid-induced ventilatory impairment (OIVI). OIVI involves suppression of the central respiratory drive, potential obstruction of the upper airway, and decreased gas exchange efficiency, all of which can exacerbate respiratory difficulties and complicate the management of these patients. It is particularly concerning that patients with OHS are more susceptible to complications associated with opioid medications, including respiratory failure and excessive sedation [97]. Approaches to pain management can involve the placement of nerve block catheters delivering local anesthetics, complemented by the use of adjunct medications such as acetaminophen and non-steroidal anti-inflammatory drugs (NSAIDs) to enhance analgesic effects [98].

## 7. Complications and Outcomes

The complications linked to OHS are diverse and significantly impact both morbidity and mortality. Individuals with this condition frequently present with notable comorbidities, including hypertension, type 2 diabetes, and cardiovascular disease, which collectively exacerbate their overall health risks [64,99,100]. Sustained adherence to PAP therapy in the long term is essential for enhancing clinical outcomes and plays a key role in effectively managing associated comorbidities in this patient group [64]. Zheng et al., in an observational study, revealed that neurocognitive functions exhibited minimal progress after three months of PAP therapy, likely due to the complex and multifactorial origins of cognitive impairment in patients with OHS [99]. Consistent monitoring and periodic adjustments to maximize efficacy and reduce potential complications faces challenges such as skin irritation from masks, air leaks, and, in certain cases, air escaping through the nasolacrimal duct and highlights the importance of continuous evaluation and customization of treatment protocols to ensure optimal comfort and effectiveness [4,10]. Additionally, the psychological impact of prolonged therapy, encompassing social influences and patient motivation, significantly affects adherence levels and overall treatment success [101].

Research shows that patients with OHS often experience significantly prolonged ICU stays, primarily because of their intricate health issues. It has been reported that the average duration of hospitalization for these patients is approximately 18.4 days, in contrast to an average of approximately 8.8 days in non-obese individuals [102]. Additionally, a study conducted by Carrillo et al. found that only 55% of OHS patients admitted to the ICU for acute hypercapnic respiratory failure received home PAP support (CPAP/NIV), emphasizing the need for regular long-term PAP home therapy after an episode of acute-on-chronic respiratory failure [103,104,105]. Recognizing these persistent health hurdles underscores the critical need for ongoing monitoring, emphasizing the significance of thorough discharge strategies and continuous supportive care for this vulnerable population [106].

## 8. Conclusions

In conclusion, Obesity Hypoventilation Syndrome (OHS) embodies a complex interplay of physiological, metabolic, and psychological factors that demand an integrated, multidisciplinary approach. Its rising prevalence underscores the urgency for early diagnosis, precise risk stratification, and personalized management strategies that encompass respiratory support, metabolic control, and behavioral interventions. While current treatments, particularly non-invasive ventilation, significantly improve patient outcomes, challenges such as long-term adherence, complication management, and addressing comorbidities remain pivotal to enhancing survival and quality of life. Continued research into the pathophysiological mechanisms and personalized therapeutic options holds promise for transforming the clinical landscape of OHS, ultimately ensuring better prognosis and comprehensive care for affected individuals. A comprehensive approach that includes both pharmacological and non-pharmacological options—supported by sustained adherence to therapy and weight management strategies, including surgical interventions—holds promise for significantly improving long-term outcomes, quality of life, and survival rates in this high-risk population.

## Data Availability

No new data were created or analyzed in this study. Data sharing is not applicable to this article.
